# Caregiver Contribution to Self-Care in Adults with Inflammatory Bowel Disease: A Cross-Sectional Multicenter Study

**DOI:** 10.3390/nursrep16040110

**Published:** 2026-03-27

**Authors:** Daniele Napolitano, Alessio Lo Cascio, Mattia Bozzetti, Fabrizio Benedetti, Giulia Petruccini, Francesco Petrosino, Silvia Cilluffo, Francesca Trotta, Davide Bartoli, Ercole Vellone, Gianluca Pucciarelli

**Affiliations:** 1 SITRA and Scientific Direction-Fondazione Policlinico Gemelli IRCCS, 00168 Rome, Italy; daniele.napolitano@policlinicogemelli.it; 2 Direction of Health Professions, La Maddalena Cancer Center, 90146 Palermo, Italy; locascio.alessio@lamaddalenanet.it; 3 Direction of Health Professions, ASST Cremona, 26100 Cremona, Italy; 4 Department of Biomedicine and Prevention, Tor Vergata University, 00133 Rome, Italy; fabribenedetti@libero.it (F.B.); giulia.petruccini@policlinicogemelli.it (G.P.); ercole.vellone@uniroma2.it (E.V.); gianluca.pucciarelli@uniroma2.it (G.P.); 5 Direction of Health Professions, ASL Salerno, 84124 Salerno, Italy; f.petrosino.75@gmail.com; 6 Department of Biomedical Sciences for Health, University of Milan, 20121 Milan, Italy; silvia.cilluffo@unimi.it; 7 AOU Sant’Andrea Hospital, 00189 Rome, Italy; francesca.trotta.91@gmail.com; 8 Interdisciplinary Department of Wellbeing, Health and Environmental Sustainability-BeSSA Department, Sapienza University of Rome, 02100 Rieti, Italy; bartoli.davide90@gmail.com; 9 Department of Nursing and Obstetrics, Wroclaw Medical University, 50-367 Wrocław, Poland

**Keywords:** caregiver contribution, caregiver self-efficacy, inflammatory bowel disease, informal care-givers, self-care

## Abstract

**Background/Objectives**: Inflammatory bowel disease (IBD) requires sustained self-care, yet patients’ ability to manage daily treatment and symptoms is often shaped by the support provided by informal caregivers. **Methods**: Guided by the Middle-Range Theory of Self-Care of Chronic Illness, this multicentre cross-sectional study described caregivers’ contributions to self-care maintenance, monitoring, and management in IBD, and compared these contributions between caregivers of patients with Crohn’s disease (CD) and those of patients with ulcerative colitis (UC). **Results**: A convenience sample of 275 caregivers of adult outpatients with IBD was recruited across multiple Italian centres. Caregiver contribution was measured using the Caregiver Contribution to Self-Care of Chronic Illness Inventory, together with caregiver self-efficacy and selected sociodemographic and clinical variables. Caregivers reported substantial involvement across all self-care domains, with significantly higher contributions to self-care maintenance among caregivers of patients with CD than among those caring for patients with UC. Monitoring and management scores were similar across groups. Regression analyses indicated disease-specific patterns, with caregiver gender, education, employment status, and patient clinical characteristics showing differential associations with self-care domains. **Conclusions**: These findings underscore the central role of caregivers in supporting self-care in IBD and suggest that structured, caregiver-focused approaches embedded in routine clinical pathways may strengthen dyadic chronic illness management.

## 1. Introduction

Inflammatory Bowel Disease (IBD) is a chronic, relapsing, remitting disorder comprising Crohn’s Disease (CD) and Ulcerative Colitis (UC) that imposes substantial burdens on patients and healthcare systems worldwide [1]. In many Western countries, the prevalence of IBD is nearing 1%, with more than 3 million individuals affected in the United States and similar trends reported in Canada and across Europe [2]. Regarding incidence, rates in the Western world have generally stabilized in recent decades, typically ranging from 12 to 26 cases per 100,000 persons per year. Although IBD is rarely fatal, it is associated with increased morbidity, reduced quality of life, and a modestly elevated risk of mortality, particularly in older adults with longstanding disease and comorbidities [2].

IBD imposes a profound multidimensional burden on patients. Patients experience a wide range of burdens that affect not only their physical health but also their psychological, social, and occupational functioning [3,4]. Common symptoms such as fatigue, abdominal pain, diarrhea, and urgency can severely limit daily activities, impair social interactions, and disrupt sleep [5,6]. The chronic and unpredictable nature of the disease often leads to emotional distress, anxiety, and depression. Intimate relationships and family dynamics may also be affected, contributing to isolation. Furthermore, many patients face challenges in maintaining employment or pursuing career goals, adding financial stress to the disease burden [7].

However, managing the IBD condition heavily depends on self-care by the patient, involving following medication routines, symptom observation, lifestyle adjustments, and prompt medical help whenever needed [8,9,10]. Symptom unpredictability, treatment side-effects, and psychosocial strain erode patients’ capacity for consistent self-care. In this context, informal caregivers, namely non-professional individuals identified by the patient as a primary source of unpaid support in daily life and disease management, play a crucial yet often underestimated role in IBD care [11,12]. In this study, the term caregiver does not refer exclusively to family members, but includes relatives, spouses or partners, friends, and other informal support persons involved in the patient’s care. This support can significantly affect how well patients care for themselves, ultimately shaping their health outcomes and overall well-being [6].

Caregivers help patients with tasks such as sticking to medication schedules, maintaining a diet, and managing symptoms [13,14]. They also provide comfort and help navigate the complexities of healthcare systems. In addition, when patients experience worsening due to illness flare-ups or feel tired or mentally strained, it becomes increasingly crucial for caregivers to support the consistency and effectiveness of self-care practices actively [15]. Studies conducted on chronic illnesses, such as diabetes and cancer, have revealed the significant impact of caregiver assistance on self-care and patient outcomes [16,17].

Within the Middle Range Theory of self-care, self-care in chronic illness is conceptualized as a dynamic process comprising self-care maintenance, self-care monitoring, and self-care management [18]. Caregivers are indispensable in helping patients maintain stability, recognize early warning signs, and manage exacerbations. Their contribution, defined as caregiver contribution to self-care [19,20,21], is not merely supportive but an active, structured process. It is distinct from caregiver burden (the strain of caregiving); contribution refers to the actual behaviours performed to support the patient. Instruments such as the Caregiver Contribution to Self-Care of Chronic Illness Inventory (CC-SC-CII) demonstrate that this engagement is measurable and serves as a critical mechanism enhancing patient outcomes, including therapy adherence and quality of life [17,22,23]. Consequently, acknowledging and reinforcing caregivers’ contributions is crucial to promoting effective and sustained self-care.

Research conducted in populations with chronic illnesses, such as heart failure, has shown that caregiver self-efficacy is a strong predictor of practical support [24]. Several elements can influence the quality and extent of caregiver support, including the caregiver’s health status, emotional resilience, knowledge of the disease, the quality of the caregiver–patient relationship, and perceived burden of care [23]. Factors related to the caregiver, such as age, gender, educational level, relationship to the patient, living arrangements, caregiving experience, and coping strategies, are all relevant [25]. Additionally, patient factors, including disease severity, cognitive abilities, and emotional health, significantly influence how caregivers assist [26,27].

In the IBD context, caregiver contributions appear to be shaped by multiple determinants. Among personal characteristics, younger age, female gender, low income, and higher education are associated with a higher burden, which is associated with lower caregiver engagement [28,29]. Clinically, more severe disease, the presence of fistulas, and a diagnosis of CD worsen the care burden, focusing more attention on lifestyle than disease management. In contrast, UC patients tend to concentrate on managing immediate symptoms such as rectal bleeding [29]. However, caregivers of patients with CD generally report higher levels of stress and burden than those of patients with UC due to greater clinical unpredictability and a frequent need for surgery [30]. The economic impact is also more significant in CD cases, leading to increased absenteeism and job presenteeism [31]. Because CD often requires dietary management and lifestyle changes (Maintenance), while UC is more frequently characterized by acute flares (Management), these clinical differences may be associated with distinct caregiving demands. In CD, caregivers are involved in more complex management due to systemic involvement and frequent complications [32]. In contrast, in UC, their role primarily focuses on promoting medication adherence and monitoring symptoms.

Although emerging evidence suggests that caregivers play an important role in supporting treatment adherence, symptom monitoring, and day-to-day disease management in IBD, the current literature remains fragmented and underdeveloped. In particular, most available studies have primarily focused on caregiver burden, psychosocial distress, or quality of life, whereas the caregiver’s active contribution to patient self-care has been much less explicitly investigated. Moreover, little is known about how caregiver contributions vary across the three dimensions of self-care maintenance, monitoring, and management, whether they differ by IBD subtype (CD vs. UC), and which caregiver- and patient-related factors are associated with greater or lower contributions. This represents an important knowledge gap, because understanding the specific patterns and determinants of caregiver contribution may help identify targets for tailored educational and supportive interventions.

The overall aim of this study was to investigate caregivers’ contributions to self-care in adults with IBD across three domains: self-care maintenance, monitoring, and management. Specifically, the study addressed the following research questions: (1) What levels of caregiver contribution to self-care maintenance, monitoring, and management are reported in adults with IBD? (2) Do these levels differ between caregivers of patients with CD and those with UC? (3) Which caregiver- and patient-related sociodemographic and clinical characteristics are associated with variations in caregiver contribution to self-care?

## 2. Materials and Methods

### 2.1. Design

A multicenter, cross-sectional study was conducted between April and June 2024. The study is registered on ClinicalTrials.gov with Identifier NCT06015789 [33].

### 2.2. Setting and Sampling

A convenience sample of consecutive caregivers of adult outpatients with IBD (CD or UC) was recruited during routine clinical visits. Caregivers were identified by patients as the primary informal, non-professional person involved in their daily care and disease management, whether a family member or another significant source of informal support. No formal a priori sample size calculation was performed for this cross-sectional analysis; however, the study was conducted within the broader IBD-SELF protocol, which planned to recruit 250 patient–caregiver dyads across nine Italian IBD centres. All participants were enrolled consecutively over the study period to reduce selection bias. The study was reported in accordance with the Strengthening the Reporting of Observational Studies in Epidemiology (STROBE) guideline (Supplementary Materials).

### 2.3. Inclusion and Exclusion Criteria

Eligible participants were primary caregivers of adult patients with a confirmed diagnosis of CD or UC. Caregivers were required to be 18 years or older and to have provided care for at least six months. Exclusion criteria included caregiving for patients with a disease duration of less than 12 months or with major coexisting chronic illnesses.

### 2.4. Instruments

Caregivers completed a structured questionnaire collecting sociodemographic information (age, gender, education, marital status, employment status, relationship to the patient, living arrangement, caregiving duration, and use of support services) and selected patient-related clinical variables (age, education, employment status, IBD type, disease duration, current therapy, prior surgery, and perianal disease). Disease activity was assessed using an integrated, disease-specific approach that combined clinical, biochemical, and endoscopic criteria. Clinical activity in CD was measured using the Harvey–Bradshaw Index (HBI), with the following categories: remission (0–4), mild (5–7), moderate (8–16), and severe (≥16). Clinical activity in UC was measured using the partial Mayo score, categorized as remission (≤2), mild (3–4), moderate (5–6), or severe (7–9). Biochemical activity was evaluated using C-reactive protein (CRP) and fecal calprotectin. Biochemical remission was defined as CRP ≤ 0.5 mg/dL and fecal calprotectin ≤ 250 μg/g, whereas values above these thresholds were considered suggestive of ongoing biochemical activity. These biomarkers were not used in isolation, but were interpreted together with disease-specific clinical scores and endoscopic findings. Endoscopic activity was evaluated using the Simple Endoscopic Score for Crohn’s Disease (SES-CD) for CD and the Mayo endoscopic subscore for UC. Based on the overall available clinical, biochemical, and endoscopic information, patients were classified as being in remission or as having mild, moderate, or severe disease activity. For analysis, these categories were further grouped into remission/mild and moderate/severe. Patient fatigue attention was recorded as a single ordinal item (ranging from 1 to 5) reflecting the caregiver’s attentiveness to recognizing and responding to signs of patient fatigue (higher values indicating greater attentiveness).

#### 2.4.1. Caregiver Contribution to Self-Care

Caregiver contribution to self-care was assessed using the Caregiver Contribution to Self-Care of Chronic Illness Inventory (CC-SC-CII) [18,20,34], an instrument that evaluates the extent to which caregivers support patients across the three components of self-care maintenance, self-care monitoring, and self-care management. Specifically, the Self-Care Maintenance scale assesses caregiver behaviours aimed at helping the patient to preserve physical and emotional stability, such as encouraging medication adherence and healthy lifestyle practices. The Self-Care Monitoring scale assesses the caregiver’s support in observing symptoms and detecting changes in health status. Finally, the Self-care management scale measures how caregivers assist patients in recognizing and responding to symptom exacerbations. Each CC-SC-CII item uses a five-point Likert scale, ranging from 1 = “never” to 5 = “always.” Standardized scores from 0 to 100 are calculated for each scale, with higher scores indicating a greater contribution to each self-care component.

#### 2.4.2. Caregiver Self-Efficacy in Contributing to Self-Care

Caregiver self-efficacy in contributing to patient self-care was assessed using the Caregiver Self-Efficacy in Contributing to patient Self-Care Scale (CSE-CSC) [35]. This 10-item valid and reliable instrument evaluates caregivers’ confidence in assisting patients with self-care maintenance, monitoring, and management [36]. Each item is scored on a 5-point Likert scale, ranging from 1 (“not confident”) to 5 (“very confident”). Responses are then standardized to a 0–100 scale, with higher scores indicating greater self-efficacy.

### 2.5. Statistical Analysis

All statistical analyses were conducted using R 4.3.3. software along with relevant R packages for data management, visualization, and statistical modelling. Descriptive analyses were conducted to summarize the sample’s characteristics. For continuous variables, normality was assessed using the Shapiro–Wilk test, along with evaluations of skewness and kurtosis, and visual inspection of residuals. Variables not normally distributed were summarized using medians and interquartile ranges (IQRs), and categorical variables were described with absolute (n) frequencies and percentages (%). Group comparisons by pathology were conducted using non-parametric Mann–Whitney U tests for continuous variables and χ^2^ or Fisher’s exact tests for categorical variables, as appropriate. To account for multiple comparisons, the Benjamini–Hochberg procedure was applied to control the false discovery rate.

Preliminary bivariate associations were explored to further characterize the relationships among study variables. Point-biserial correlations were computed to examine the associations between continuous outcomes and dichotomous variables. Spearman’s rank-order correlations were calculated for ordinal or multinomial predictors with three or more levels. Additionally, Kruskal–Wallis tests were employed to assess differences in self-care domains across categorical variables with multiple levels. When Kruskal–Wallis tests revealed significant overall effects, Dunn’s post hoc tests with Bonferroni correction were conducted to identify pairwise group differences.

Linear regression models were used to examine associations between caregiver- and patient-related predictors and the three domains of the CC-SC-CII: Maintenance, Monitoring, and Management. Interaction terms between each predictor and pathology were included to test whether the strength or direction of associations differed between caregivers of patients with CD and UC. This approach allowed the identification of effect modifications by pathology—i.e., whether certain predictors had a stronger or weaker association with CC-SC-CII outcomes in one condition versus the other.

Predictors were included either as continuous or categorical variables, depending on their nature. Some categorical predictors with more than two levels were dichotomized to improve model interpretability and reduce overfitting, particularly when certain levels had sparse data. For example, “Marital status” was recoded into *Married* vs. *Other*, and “Education” into *High* (high school or higher) vs. *Low* (middle school or lower). All selected predictors were retained in the models to allow consistent comparison of effect estimates across pathologies and self-care domains. Model residuals were checked to ensure that the assumptions of linearity, homoscedasticity, and normality were met sufficiently to justify the use of linear models without data transformation.

## 3. Results

A total of 275 caregivers were enrolled in the study. The study is evenly split between caregivers of patients with CD (47.6%) and patients with UC (52.4%), with a median age of 43.5 years. The gender distribution of caregivers was slightly skewed toward female participants, with 58.2% identifying as female and 41.8% as male. The socio-demographic characteristics of the sample are presented in Table 1.

The analysis revealed that CSE-CSC scores were similar between individuals in the caregiver group with CD and those in the UC group. Specifically, the CD group reported a median [interquartile range] of 75.0 [60.0–85.0], while the UC group reported 75.0 [65.0–81.2] (*p* = 0.343). Similarly, caregiver burden scores did not differ significantly between the groups, with a median [IQR] of 12.0 [6.0–19.2] in the CD group and 12.0 [6.0–19.0] in the UC group (*p* = 0.657).

### 3.1. Description of CC-SC-CII Items

The analysis of individual CC-SC-CII items is presented in Table 2. In CC-SC-CII Maintenance, caregivers reported high adherence to medical recommendations, such as attending routine healthcare appointments (median = 4.0) and taking prescribed medications (median = 4.0). However, behaviours such as engaging in physical activity (median = 3.0) and managing stress (median = 3.0) received lower scores. Notably, significant differences between caregiver of patients with CD and patients with UC were observed in items such as avoiding illness (*p* = 0.003), physical activity (*p* = 0.001), dietary management (*p* = 0.024), attending appointments (*p* = 0.026), medication adherence (*p* = 0.047), and stress relief (*p* = 0.016), with CD caregivers generally reporting higher engagement.

In CC-SC-CII Monitoring, the most frequent behaviours were paying attention to changes in the person’s feelings (median = 5.0) and monitoring for symptoms (median = 4.0).

In CC-SC-CII Management, the most common behaviours were reporting symptoms to the healthcare provider during office visits (median = 4.0) and recognizing symptoms promptly (median = 5.0). Other behaviours, such as recommending rest or dietary changes to alleviate symptoms, received lower scores (both medians = 3.0) (Table 2).

Item 13 serves as a conceptual bridge between CC-SC-CII monitoring and CC-SC-CII management. It asks how quickly the caregiver recognized a recent symptom related to the patient’s health condition. This item received high scores (median = 5.0), with no significant difference between the CD and UC groups (*p* = 0.684).

### 3.2. Caregiver Contribution to Self-Care Scores

**Figure 1:**
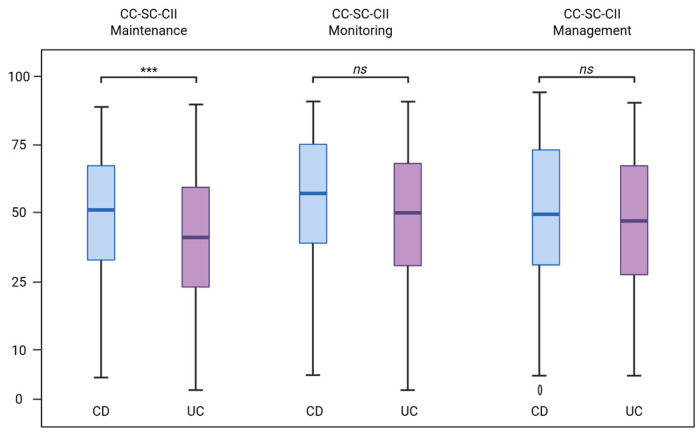
Caregiver contribution to self-care differences between CD and UC. Notes: CC-SC-CII, Caregiver Contribution to Self-Care of Chronic Illness Inventory; CD, Crohn’s disease; UC, ulcerative colitis. Group differences were tested using the Mann–Whitney U test. Points represent individual observations; the central line indicates the median. Sign. Codes: *** at *p* < 0.001; ns = not significant.

The total CC-SC-CII Maintenance score was significantly higher among caregivers of patients with CD than among caregivers of patients with UC (68.0 [44.5–79.0] vs. 52.0 [32.0–71.0], *p* = 0.001). No significant differences were observed between the two groups in CC-SC-CII Monitoring scores (80.0 [55.0–97.5] vs. 75.0 [45.0–95.0], *p* = 0.203) or CC-SC-CII Management scores (58.0 [46.0–75.0] vs. 58.0 [38.0–75.0], *p* = 0.384) (Figure 1).

### 3.3. Differences in Caregiver Contribution to Self-Care

No significant differences in CC-SC-CII domains emerged across patient-related categorical variables. Specifically, Kruskal–Wallis tests showed no significant associations between patient education, occupation, therapy, previous surgeries, or perianal disease and any of the three self-care dimensions.

For CC-SC-CII Maintenance, no significant differences were found across Education level, Marital status, Occupation, Relationship with the patient, Care activities impact on quality of life, Care load, Daily activities, Support services accessed, or Time in charge.

For CC-SC-CII Monitoring, a significant difference emerged across Occupation groups (χ^2^_(4)_ = 11.579, *p* = 0.021). Post hoc Dunn tests indicated that unemployed caregivers had significantly lower contributions to monitoring behaviours compared to workers (*p* = 0.038).

For CC-SC-CII Management, significant differences were detected across Occupation (χ^2^_(4)_ = 9.734, *p* = 0.045) and Care load (χ^2^_(3)_ = 13.519, *p* = 0.004). Post hoc analyses revealed that retired caregivers had significantly lower self-care management scores than homemakers (*p* = 0.030) and that workers had significantly lower scores than retired individuals (*p* = 0.037). Regarding Care load, caregivers with moderate load showed significantly lower self-care management scores than those with mild load (*p* = 0.002).

### 3.4. Correlations

Point-biserial correlations were calculated to examine associations between CC-SC-CII dimensions and dichotomous variables. For CC-SC-CII Maintenance and Monitoring no significant observations were observed. For CC-SC-CII Management, Gender was negatively and significantly associated with the outcome (r = −0.186, *p* = 0.0020), indicating that male gender was associated with lower management scores. Additionally, Minor Children showed a positive and significant association with CC-SC-CII Management (r = 0.144, *p* = 0.0172), (Table 3).

### 3.5. Regression Analysis

The regression model for CC-SC-CII Maintenance was not statistically significant (R^2^ = 0.034). Within the UC group, higher maintenance scores were associated with female gender (β = 15.80, SE = 6.67, *p* = 0.019) and longer disease duration (β = 0.81, SE = 0.40, *p* = 0.044). No other significant associations were observed.

The regression model for CC-SC-CII Monitoring was statistically significant (R^2^ = 0.039). In the CD group, higher monitoring scores were associated with caregiver occupation (worker vs. other: β = −14.48, SE = 6.97, *p* = 0.039). In the UC group, higher monitoring scores were associated with the absence of previous surgery (β = −21.59, SE = 9.60, *p* = 0.025).

The regression model for CC-SC-CII Management was statistically significant (R^2^ = 0.080). In the CD group, higher management scores were associated with previous surgery (β = 12.31, SE = 5.11, *p* = 0.017). In the UC group, higher management scores were associated with female gender (β = 14.07, SE = 4.02, *p* = 0.001) and younger patient age (β = −0.58, SE = 0.28, *p* = 0.037). The regression results are reported in Table 4.

## 4. Discussion

This multicentre cross-sectional study provides novel evidence into the contribution of informal caregivers to self-care in patients with IBD, highlighting significant differences between CD and UC and patterns of association with selected sociodemographic and caregiving-related variables. Guided by the Middle-Range Theory of Self-Care of Chronic Illness [18,19], the findings indicate that caregiver involvement spans all three self-care domains, maintenance, monitoring, and management, and that its expression is shaped by both contextual and individual factors.

More importantly, this study extends the existing IBD literature by examining caregiver contribution not only as a generic supportive role, but as a multidimensional construct that can be described across specific self-care domains and explored in relation to disease subtype and caregiving characteristics. A key finding of this study is that caregiver contribution was not uniform across self-care domains. Caregivers of patients with CD reported greater involvement in self-care maintenance than caregivers of patients with UC, whereas no significant between-group differences emerged for monitoring and management. This pattern may reflect the distinct clinical demands of the two IBD subtypes. CD often requires more continuous support related to dietary adaptation, symptom prevention, and long-term lifestyle regulation, while UC may be perceived as more episodic, with caregiver engagement becoming more salient during acute exacerbations rather than in routine maintenance [37,38,39,40,41].

These findings are consistent with previous evidence from other chronic illnesses, such as heart failure and COPD, where caregivers demonstrate consistent engagement in symptom monitoring and acute management, but often exhibit less involvement in lifestyle-promoting behaviours [42,43].

The item-level analysis of the CC-SC-CII revealed high engagement in clinically oriented tasks, such as ensuring medication adherence, recognizing symptoms, and facilitating healthcare visits. At the same time, lower scores were reported in lifestyle domains such as promoting physical activity or stress relief. This pattern reflects a tendency observed across chronic illness contexts: caregivers often prioritize concrete, medically oriented responsibilities over behavioural or psychosocial aspects of care [24]. This finding is important because it suggests that caregiver contribution may be selectively concentrated on tasks perceived as urgent, tangible, or clinically legitimized, whereas supportive behaviours related to wellbeing, coping, and everyday health promotion may receive less attention. Similar patterns have been described in other chronic illness populations, where caregivers are more confident in responding to symptoms and treatment-related needs than in sustaining broader behavioural and psychosocial self-care processes [44,45].

Caregiver characteristics, including gender, occupation, marital status, caregiving load, and the presence of minor children, were associated with distinct patterns of support, especially in the monitoring and management domains. For instance, male gender and unemployment were negatively associated with caregiver contribution, echoing previous research showing that male caregivers may report lower confidence and practical engagement in caregiving tasks [46]. In the UC group, being female was associated with higher contributions to both monitoring and management, while being married was positively associated with maintenance. These findings may suggest that gendered expectations and social support structures may facilitate caregiving involvement in certain domains. Conversely, increasing caregiver age was negatively associated with management engagement, possibly reflecting physical or cognitive limitations that affect acute responsiveness. An unexpected finding was the positive bivariate association between caregiver burden and monitoring/management scores.

Although this finding contrasts with studies suggesting that higher burden undermines caregiving effectiveness [15,29], it should be interpreted cautiously. Because the present analysis is cross-sectional and primarily exploratory, reverse causality and bidirectional dynamics are plausible: caregivers who are more involved in monitoring and management may also perceive greater burden precisely because they assume a more active role. Rather than indicating a beneficial effect of burden, this result may reflect the intensity and complexity of caregiving work in IBD. Longitudinal and dyadic studies are needed to clarify the temporal and relational mechanisms underlying this association.

Regression models further illustrated these patterns by identifying pathology-specific associations. For example, in the CD group, prior patient surgery was associated with greater caregiver contribution to management, a plausible reflection of increased caregiver vigilance postoperatively. In the UC group, female gender and younger age were associated with greater caregiver support in the management domain. However, given the modest proportion of variance explained by the models, these findings should be interpreted as indicative of relational patterns rather than as evidence of robust or clinically actionable predictors.

These findings support the Middle-Range Theory of Self-Care, which conceptualizes caregiver contribution as an essential component of chronic illness management [19]. Importantly, the results underscore the dyadic nature of self-care: caregivers and patients function as interdependent units whose interactions determine care quality and outcomes [22]. Consequently, caregiver engagement should be considered not as ancillary, but as integral to disease management.

The present study also contributes to the literature by shifting attention from caregiver burden alone to caregiver contribution as a distinct and measurable component of IBD care. This distinction is clinically and conceptually relevant because burden captures the strain associated with caregiving, whereas contribution reflects the actual supportive behaviours that may influence patient self-care and disease management. From a clinical and organizational perspective, these findings support the value of a more holistic, person-centred approach to IBD care, in which caregiver-oriented assessment and support are integrated into routine care pathways. Tailored education, psychosocial resources, and multidisciplinary interventions may be particularly relevant for caregivers at risk of lower engagement, although these implications should be regarded as preliminary and hypothesis-generating rather than immediately prescriptive. The low use of support services observed in our sample further suggests that caregiver needs may remain under-recognized within current models of IBD care [34,47,48].

### 4.1. Strengths and Limitations

This study’s strengths include its multicentre design, relatively large and balanced sample, and the use of validated tools (CC-SC-CII and CSE-CSC). Furthermore, the inclusion of item-level analyses and disease-specific subgroup comparisons offers nuanced insights rarely addressed in the IBD literature. Another strength is the theory-informed focus on caregiver contributions across the three domains of self-care, which provides a more granular understanding of caregiving behaviours than studies focusing exclusively on burden or distress. However, several limitations must be acknowledged. The cross-sectional nature of the study precludes causal inferences and limits understanding of temporal dynamics in caregiver involvement. The reliance on self-reported data introduces potential bias, especially for socially desirable behaviours. The study also did not include psychological variables, such as mutuality, resilience, or emotional distress, which likely play a critical role in shaping caregiver behaviour. Finally, the decision to dichotomize some predictors, though analytically pragmatic, may have reduced model sensitivity and masked more subtle relationships.

Moreover, the limited explanatory power of the regression models suggests that important determinants of caregiver contribution may not have been fully captured, underscoring the need for more comprehensive, theory-informed modelling approaches in future research. Taken together, these strengths and limitations suggest that the findings should be interpreted as robust descriptive and exploratory evidence, while caution is warranted when drawing causal or clinically definitive conclusions.

### 4.2. Implications for Research and Practice

Future research should adopt a longitudinal, dyadic approach to investigate how caregiver contributions evolve over the course of the disease and with care experience. Dyadic analysis models such as the Actor-Partner Interdependence Model (APIM) could better capture the reciprocal influences between patient and caregiver behaviours. Moreover, through interviews and focus groups, qualitative research can help illuminate caregivers’ subjective experiences, such as their sense of invisibility, burden, or empowerment. From a practice standpoint, embedding caregiver assessment into routine clinical workflows and electronic health records could facilitate early identification of needs and risk factors. However, evidence from longitudinal and interventional studies is needed before specific caregiver-targeted strategies can be broadly implemented in clinical practice. Interdisciplinary teams involving IBD nurses, psychologists, and social workers should be mobilized to deliver personalized support strategies that consider clinical and relational care dimensions.

## 5. Conclusions

This study contributes to the growing literature on chronic illness care by showing that informal caregivers play a meaningful role in supporting patients with IBD in self-care across the domains of maintenance, monitoring, and management. The findings suggest that caregiver contributions are not uniform but may vary by disease subtype and selected caregiving and patient-related characteristics. By examining caregiver contributions as a distinct, multidimensional construct rather than focusing exclusively on caregiver burden, this study offers a more nuanced understanding of caregiving in IBD. Although the observed associations should be interpreted with caution, given the cross-sectional design and the modest explanatory power of some models, the findings suggest that caregiver-focused strategies such as tailored education, symptom-monitoring support, and psychosocial resources may represent promising areas for future intervention. In this context, a dyadic perspective may be particularly valuable, as it conceptualizes patient and caregiver as interdependent partners in self-care rather than as separate actors. This approach may help inform interventions not only to strengthen self-care processes but also to support caregiver sustainability over time. Future longitudinal and interventional studies are needed to clarify which caregiver-targeted strategies are most effective in improving outcomes for both patients and caregivers.

## Figures and Tables

**Table 1:** Socio-demographic characteristics of caregivers.

Variables	Caregiver of a Patient with CD (n = 131; 47.6%)	Caregiver of a Patient with UC (n = 144; 52.4%)	*p*-Value
**Gender n (%)**			
Female	76 (58.0%)	84 (58.3%)	1.000
Male	55 (42.0%)	60 (41.7%)	1.000
**Age of Caregiver (years) Me [IQR]**	50.0 [40.5–59.0]	53.5 [44.0–60.0]	0.200
**Age of Patient (years) Me [IQR]**	44.0 [28.0–58.0]	39.5 [28.0–55.0]	0.515
**Years since diagnosis**	8.0 [4.0–13.0]	10.0 [5.0–17.5]	0.058
**Patient’s Disease Activity n (%)**			
Remission	98 (74.8%)	51 (35.4%)	**<0.0001**
Mild	14 (10.7%)	24 (16.7%)	
Moderate	12 (9.2%)	34 (23.6%)	
Severe	7 (5.3%)	35 (24.3%)	
**Current therapy**			0.068
Biological therapy	115 (88.5%)	131 (90.9%)	
No Biological therapy	14 (10.8%)	13 (9.0%)	
**Previous surgery**			
>1 Time	23 (17.7%)	23 (16.0%)	0.123
1	15 (11.5%)	30 (20.8%)	
Never	91 (70.0%)	91 (63.2%)	
**Perianal disease n (%)**			
No	106 (81.5%)	129 (89.6%)	0.112
Yes	23 (17.7%)	15 (10.4%)	
**Patient Fatigue Attention Me [IQR]**	2.0 [1.0–2.0]	2.0 [2.0–3.0]	0.299
**Educational Level of Caregiver n (%)**			
Bachelor’s degree	29 (22.1%)	44 (30.6%)	0.379
High School	66 (50.4%)	68 (47.2%)	
Middle School	33 (25.2%)	28 (19.4%)	
Primary School	3 (2.3%)	4 (2.8%)	
**Educational Level of Patient n (%)**			
Bachelor’s degree	35 (26.9%)	48 (33.3%)	0.654
Primary school	4 (3.1%)	5 (3.5%)	
High school	62 (47.7%)	60 (41.7%)	
Middle school	28 (21.5%)	31 (21.5%)	
**Marital status of Caregiver n (%)**			
Divorced	10 (7.6%)	5 (3.5%)	0.486
Engaged	12 (9.2%)	11 (7.6%)	
Married	96 (73.3%)	117 (81.2%)	
Single	11 (8.4%)	9 (6.2%)	
Widowed	2 (1.5%)	2 (1.4%)	
**Sons of Caregiver (Minor children) n (%)**			
No	98 (74.8%)	114 (79.2%)	0.475
Yes	33 (25.2%)	30 (20.8%)	
**Occupation of Caregiver n (%)**			
Homemaker	15 (11.5%)	14 (9.7%)	0.251
Retired	21 (16.0%)	25 (17.4%)	
Student	6 (4.6%)	2 (1.4%)	
Unemployed	6 (4.6%)	2 (1.4%)	
Active worker	83 (63.4%)	101 (70.1%)	
**Occupation of patient n (%)**			
Active worker	81 (62.3%)	90 (62.5%)	0.354
Homemaker	9 (6.9%)	8 (5.6%)	
Retired	14 (10.8%)	17 (11.8%)	
Student	16 (12.3%)	19 (13.2%)	
Unemployed	9 (6.9%)	10 (6.9%)	
**Relationship with patient n (%)**			
Friend	18 (13.7%)	11 (7.6%)	0.239
None	12 (9.2%)	11 (7.6%)	
Parent	20 (15.3%)	23 (16.0%)	
Partner	5 (3.8%)	7 (4.9%)	
Relative	12 (9.2%)	14 (9.7%)	
Sibling	6 (4.6%)	1 (0.7%)	
Spouse	58 (44.3%)	77 (53.5%)	
**Caregiving Load n (%)**			
Mild	54 (41.2%)	49 (34.0%)	0.469
Moderate	60 (45.8%)	76 (52.8%)	
Severe	17 (13.0%)	18 (12.5%)	
**Daily activities of patient n (%)**			
Intense activity	35 (26.7%)	29 (20.1%)	0.213
Moderate activity	71 (54.2%)	93 (64.6%)	
Sedentary	25 (19.1%)	22 (15.3%)	
**Smoking Status n (%)**			
Ex smoker	28 (21.4%)	25 (17.4%)	0.620
Nonsmoker	74 (56.5%)	89 (61.8%)	
Smoker	29 (22.1%)	30 (20.8%)	
**Support service n (%)**			
Gastroenterologist	96 (73.3%)	91 (63.2%)	**0.012**
General Practitioner	15 (11.5%)	12 (8.3%)	
Internet	3 (2.3%)	4 (2.8%)	
Nurses	10 (7.6%)	8 (5.6%)	
Other	6 (4.6%)	24 (16.7%)	
Volunteer Associations	1 (0.8%)	5 (3.5%)	
**Time in charge n (%)**			
>1 Year	21 (16.0%)	29 (20.1%)	0.607
>3 Years	18 (13.7%)	22 (15.3%)	
>5 Years	63 (48.1%)	58 (40.3%)	
Months	29 (22.1%)	35 (24.3%)	

Notes: Bold Values are statistically significant. CD, Crohn’s Disease; UC, Ulcerative Colitis; Me, Median; IQR, Interquartile Range.

**Table 2:** Descriptive statistics of CC-SC-CII items and their comparison between CD and UC.

Items	Total Caregiver	Caregiver of Patient with CD (n = 131; 47.6%)	Caregiver of Patient with UC (n = 144; 52.4%)	*p*-Value
**CC-SC-CII Maintenance**	**57.0 [36.0–75.0]**	**68.0 [44.5–79.0]**	**52.0 [32.0–71.0]**	**0.001**
Make sure to get enough sleep? (Item 1)	3.0 [1.0–4.0]	3.0 [2.0–4.0]	3.0 [1.0–4.0]	0.340
Try to avoid getting sick (e.g., flu shot, wash your hands)? (Item 2)	4.0 [2.0–5.0]	4.0 [2.5–5.0]	3.0 [2.0–4.0]	**0.003**
Do physical activity (e.g., take a brisk walk, use the stairs)? (Item 3)	3.0 [2.0–4.0]	3.0 [3.0–4.0]	3.0 [2.0–4.0]	**0.001**
Eat special foods or avoid certain foods? (Item 4)	4.0 [3.0–5.0]	4.0 [3.0–5.0]	4.0 [3.0–5.0]	**0.024**
Keep appointments for routine or regular health care? (Item 5)	4.0 [2.0–5.0]	5.0 [2.0–5.0]	4.0 [1.0–5.0]	**0.026**
Take prescribed medicines without missing a dose? (Item 6)	4.0 [2.0–5.0]	4.0 [2.0–5.0]	3.0 [1.0–5.0]	**0.047**
Do something to relieve stress (e.g., mindfulness, yoga, music)? (Item 7)	3.0 [1.0–4.0]	3.0 [2.0–5.0]	3.0 [1.0–4.0]	**0.016**
**CC-SC-CII Monitoring**	**80.0 [50.0–95.0]**	**80.0 [55.0–97.5]**	**75.0 [45.0–95.0]**	0.203
Monitor the health condition? (Item 8)	4.0 [3.0–5.0]	4.0 [3.0–5.0]	4.0 [3.0–5.0]	0.134
Monitor for medication side-effects? (Item 9)	4.0 [2.0–5.0]	4.0 [2.5–5.0]	3.5 [2.0–5.0]	0.214
Pay attention to changes in how one feels? (Item 10)	5.0 [3.0–5.0]	5.0 [3.5–5.0]	4.0 [3.0–5.0]	0.221
Monitor whether one tires more than usual doing normal activities? (Item 11)	4.0 [3.0–5.0]	4.0 [3.0–5.0]	4.0 [3.0–5.0]	0.435
Monitor for symptoms? (Item 12)	4.0 [3.0–5.0]	4.0 [3.0–5.0]	4.0 [3.0–5.0]	0.535
**CC-SC-CII Management**	**58.0 [42.0–75.0]**	**58.0 [46.0–75.0]**	**58.0 [38.0–75.0]**	0.384
^§^ Many patients have symptoms due to their health condition or due to the treatment they receive for it. The last time the person you care for had a symptom, how quickly did you recognize it as a symptom of health condition of the person you care for? (Item 13)	5.0 [3.0–6.0]	5.0 [4.0–6.0]	5.0 [3.0–6.0]	0.684
Change what the person you care for eats or drinks to make the symptom decrease or go away? (Item 14)	3.0 [2.5–5.0]	3.0 [3.0–5.0]	3.0 [2.0–5.0]	0.388
Recommend the person you care for to change the activity level (e.g., slow down, rest)? (Item 15)	3.0 [2.0–4.0]	3.0 [3.0–4.5]	3.0 [2.0–4.0]	0.171
Recommend the person you care for to take a medicine to make the symptom decrease or go away? (Item 16)	3.0 [1.0–4.0]	3.0 [1.0–4.0]	3.0 [1.0–4.0]	0.319
Tell about the symptom to the healthcare provider of the person you care for at the next office visit? (Item 17)	4.0 [3.0–5.0]	4.0 [3.0–5.0]	4.0 [3.0–5.0]	0.165
Call the healthcare provider of the person you care for to get guidance? (Item 18)	3.0 [2.0–5.0]	3.0 [2.0–5.0]	3.0 [2.0–5.0]	0.240
Think of a treatment you used the last time the person you care for had symptoms. Did the treatment you used make him/her feel better? (Item 19)	3.0 [3.0–4.0]	3.0 [3.0–4.0]	3.0 [2.0–4.0]	0.716

Notes. Bold values are statistically significant. CC-SC-CII, Caregiver Contribution to Self-Care of Chronic Illness Inventory; CD, Crohn’s Disease; UC, Ulcerative Colitis. ^§^ Bridge item.

**Table 3:** Correlation matrix.

	Age (Caregiver)	Age (Patient)	Years from Diagnosis	CC-SC-CII Maintenance	CC-SC-CII Monitoring	CC-SC-CII Management
Age (Caregiver)	–					
Age (Patient)	−0.01	–				
Years from Diagnosis	−0.01	0.30 ***	–			
CC-SC-CII Maintenance	−0.04	0.02	−0.03	–		
CC-SC-CII Monitoring	−0.00	0.00	−0.03	0.34 ***	–	
CC-SC-CII Management	−0.11.	0.05	−0.00	0.32 ***	0.39 ***	–

Note. Sign. Codes: ‘.’0.1; ’***’ 0.001; Bold values are statistically significant. CC-SC-CII, Caregiver Contribution to Self-Care of Chronic Illness Inventory.

**Table 4:** Multivariable linear regression analyses for caregiver contribution to self-care domains.

(**A**)**. CC-SC-CII Maintenance**		
**Variables (reference group)**	**CD B (SE), *p*-value**	**UC B (SE), *p*-value**
Intercept	**48.35 (19.11), 0.012**	**47.00 (20.87), 0.026**
Caregiver gender (female)	−9.17 (4.91), 0.063	6.63 (4.52), 0.145
Caregiver education (high)	7.50 (5.74), 0.194	7.04 (5.63), 0.213
Caregiver occupation (worker)	−6.59 (5.42), 0.227	−5.83 (5.62), 0.302
Surgery history (>1)	4.43 (5.81), 0.447	1.68 (4.69), 0.722
Patient age	0.19 (0.15), 0.198	−0.22 (0.16), 0.182
Years since diagnosis	−0.33 (0.30), 0.278	0.48 (0.26), 0.069
R^2^	0.034	
(**B**). **CC-SC-CII Monitoring**		
**Variables (reference group)**	**CD B (SE), *p*-value**	**UC B (SE), *p*-value**
Intercept	**85.77 (24.57), 0.001**	**75.26 (26.82), <0.001**
Caregiver gender (female)	−0.47 (6.31), 0.941	**12.60 (5.81), 0.032**
Caregiver education (high)	13.79 (7.37), 0.063	4.60 (7.24), 0.526
Caregiver occupation (worker)	−**14.48 (6.97), 0.039**	−1.63 (7.22), 0.821
Surgery history (>1)	10.77 (7.47), 0.152	−10.82 (6.03), 0.075
Patient age	−0.05 (0.19), 0.809	−0.06 (0.21), 0.758
Years since diagnosis	−0.51 (0.39), 0.193	0.27 (0.33), 0.414
R^2^	0.039	
(**C**). **CC-SC-CII Management**		
**Variables (reference group)**	**CD B (SE), *p*-value**	**UC B (SE), *p*-value**
Intercept	26.95 (16.60), 0.107	**58.81 (18.57), 0.002**
Caregiver gender (female)	4.26 (4.26), 0.319	**14.07 (4.02), 0.001**
Caregiver education (high)	0.82 (4.98), 0.870	−1.21 (5.01), 0.810
Caregiver occupation (worker)	2.27 (4.71), 0.630	6.97 (5.00), 0.166
Surgery history (>1)	**12.31 (5.11), 0.017**	−1.95 (4.18), 0.641
Patient age	0.13 (0.13), 0.326	−0.04 (0.14), 0.763
Years since diagnosis	0.02 (0.26), 0.946	−0.05 (0.23), 0.820
R^2^	0.080	

Note. Values are presented as B (SE) and *p*-values from separate multivariable linear regression models. CC-SC-CII = Caregiver Contribution to Self-Care of Chronic Illness Inventory; CD = Crohn’s disease; UC = ulcerative colitis. Statistically significant associations (*p* < 0.05) are shown in bold.

## Data Availability

The data presented in this study are available on request from the corresponding author. The data are not publicly available due to privacy and ethical restrictions.

## Supplementary Materials

The following supporting information can be downloaded at https://www.mdpi.com/article/10.3390/nursrep16040110/s1; STROBE Statement—checklist of items that should be included in reports of observational studies.

## Public Involvement Statement

No public involvement in any aspect of this research.

## Guidelines and Standards Statement

This manuscript was drafted against the STROBE (Strengthening the Reporting of Observational Studies in Epidemiology) for observational research.

## Abbreviations

The following abbreviations are used in this manuscript:

APIM	Actor-Partner Interdependence Model
CC-SC-CII	Caregiver Contribution to Self-Care of Chronic Illness Inventory
CD	Crohn’s Disease
CI	Confidence Interval
CSE-CSC	Caregiver Self-Efficacy in Contributing to Patient Self-Care
IBD	Inflammatory Bowel Disease
IQR	Interquartile Range
STROBE	Strengthening the Reporting of Observational Studies in Epidemiology
UC	Ulcerative Colitis

## References

[1] Lamb C.A., Kennedy N.A., Raine T., Hendy P.A., Smith P.J., Limdi J.K., Hayee B., Lomer M.C.E., Parkes G.C., Selinger C. (2019). British Society of Gastroenterology consensus guidelines on the management of inflammatory bowel disease in adults. Gut.

[2] Kaplan G.G., Windsor J.W. (2021). The four epidemiological stages in the global evolution of inflammatory bowel disease. Nat. Rev. Gastroenterol. Hepatol..

[3] Lönnfors S., Vermeire S., Greco M., Hommes D., Bell C., Avedano L. (2014). IBD and health-related quality of life—Discovering the true impact. J. Crohns Colitis.

[4] Zhang J., Liu C., An P., Chen M., Wei Y., Li J., Zeng S., Xiang D., Cai Y., Li J. (2024). Psychological symptoms and quality of life in patients with inflammatory bowel disease in China: A multicenter study. United Eur. Gastroenterol. J..

[5] Burisch J., Hart A., Sturm A., Rudolph C., Meadows R., Jus A., Dawod F., Patel H., Armuzzi A. (2025). Residual Disease Burden Among European Patients with Inflammatory Bowel Disease: A Real-World Survey. Inflamm. Bowel Dis..

[6] Spagnuolo R., Corea A., Napolitano D., Nisticò E., Pagnotta R., Pagliuso C., Schiavoni E., Turchini L., Fiorino G., Radice S. (2021). Nursing-sensitive outcomes in adult inflammatory bowel disease: A systematic review. J. Adv. Nurs..

[7] Bernabeu P., Belén-Galipienso O., van-der Hofstadt C., Gutiérrez A., Madero-Velázquez L., García Del Castillo G., García-Sepulcre M.-F., Aguas M., Zapater P., Rodríguez-Marín J. (2024). Psychological burden and quality of life in newly diagnosed inflammatory bowel disease patients. Front. Psychol..

[8] Iizawa M., Hirose L., Nunotani M., Nakashoji M., Tairaka A., Fernandez J.L. (2023). A Systematic Review of Self-Management Interventions for Patients with Inflammatory Bowel Disease. Inflamm. Intest. Dis..

[9] Sheehan J.L., Greene-Higgs L., Resnicow K., Patel M.R., Barnes E.L., Waljee A.K., Higgins P.D.R., Cohen-Mekelburg S. (2024). Self-Efficacy, Patient Activation, and the Burden of Inflammatory Bowel Disease on Patients’ Daily Lives. Dig. Dis. Sci..

[10] Plevinsky J.M., Greenley R.N., Fishman L.N. (2016). Self-management in patients with inflammatory bowel disease: Strategies, outcomes, and integration into clinical care. Clin. Exp. Gastroenterol..

[11] Karadag P., Morris B., Woolfall K. (2022). The information and support needs of patients living with inflammatory bowel disease: A qualitative study. Chronic Illn..

[12] Delen S., Jaghult S., Blumenstein I., Pouillon L., Bossuyt P. (2024). Framework of IBD Care Delivery Across Ages. J. Crohns Colitis.

[13] Ribaudi E., Amato S., Becherucci G., Carillo S., Covello C., Mora V., Mentella M.C., Scaldaferri F., Gasbarrini A., Fanali C. (2025). Addressing Nutritional Knowledge Gaps in Inflammatory Bowel Disease: A Scoping Review. Nutrients.

[14] Volpato E., Bosio C., Previtali E., Leone S., Armuzzi A., Pagnini F., Graffigna G. (2021). The evolution of IBD perceived engagement and care needs across the life-cycle: A scoping review. BMC Gastroenterol..

[15] Thakur E., Bradford A., Hou J. (2017). Caregiver Burden in Adults with Inflammatory Bowel Disease. Clin. Gastroenterol. Hepatol..

[16] Taurisano P., De Feudis R.L., Graziano G., Marzano N., Curci A., Fidanzio A., Annunziata M.A., Antinone V., Brovelli S., Carone M. (2023). Patient-caregiver relationship in cancer fatigue and distress. A dyadic approach. Curr. Psychol..

[17] Iovino P., Lyons K.S., De Maria M., Vellone E., Ausili D., Lee C.S., Riegel B., Matarese M. (2021). Patient and caregiver contributions to self-care in multiple chronic conditions: A multilevel modelling analysis. Int. J. Nurs. Stud..

[18] Riegel B., Jaarsma T., Strömberg A. (2012). A middle-range theory of self-care of chronic illness. ANS Adv. Nurs. Sci..

[19] Vellone E., Riegel B., Alvaro R. (2019). A Situation-Specific Theory of Caregiver Contributions to Heart Failure Self-care. J. Cardiovasc. Nurs..

[20] Vellone E., Lorini S., Ausili D., Alvaro R., Di Mauro S., De Marinis M.G., Matarese M., De Maria M. (2020). Psychometric characteristics of the caregiver contribution to self-care of chronic illness inventory. J. Adv. Nurs..

[21] Riegel B., Jaarsma T., Stromberg A. (2025). Designing Interventions to Promote Self-Care. Inferm. J..

[22] De Maria M., Ausili D., Lorini S., Vellone E., Riegel B., Matarese M. (2022). Patient Self-Care and Caregiver Contribution to Patient Self-Care of Chronic Conditions: What Is Dyadic and What It Is Not. Value Health.

[23] Napolitano D., Bozzetti M., Lo Cascio A., De Stefano G., Orgiana N., Lopetuso L.R., D’Onofrio A.M., Camardese G., Papa A., Scaldaferri F. (2025). Resilience and Self-Care in Patients with Inflammatory Bowel Disease: A Multicentre Cross-Sectional Study in Outpatient Settings. J. Clin. Med..

[24] Dellafiore F., Chung M.L., Alvaro R., Durante A., Colaceci S., Vellone E., Pucciarelli G. (2019). The Association Between Mutuality, Anxiety, and Depression in Heart Failure Patient-Caregiver Dyads: An Actor-Partner Interdependence Model Analysis. J. Cardiovasc. Nurs..

[25] Loo Y.X., Yan S., Low L.L. (2022). Caregiver burden and its prevalence, measurement scales, predictive factors and impact: A review with an Asian perspective. Singapore Med. J..

[26] Huang Y., Hu J., Xie T., Jiang Z., Ding W., Mao B., Hou L. (2023). Effects of home-based chronic wound care training for patients and caregivers: A systematic review. Int. Wound J..

[27] King A., Ringel J.B., Safford M.M., Riffin C., Adelman R., Roth D.L., Sterling M.R. (2021). Association Between Caregiver Strain and Self-Care Among Caregivers with Diabetes. JAMA Netw. Open.

[28] Parekh N.K., Shah S., McMaster K., Speziale A., Yun L., Nguyen D.L., Melmed G., Kane S. (2017). Effects of caregiver burden on quality of life and coping strategies utilized by caregivers of adult patients with inflammatory bowel disease. Ann. Gastroenterol..

[29] Zand A., Kim B.J., van Deen W.K., Stokes Z., Platt A., O’Hara S., Khong H., Hommes D.W. (2020). The effects of inflammatory bowel disease on caregivers: Significant burden and loss of productivity. BMC Health Serv. Res..

[30] Zhou M., Wang M., Luo D., Sun C., Bian Q., Xu J., Lin Z. (2024). The mediating role of resilience between caregiver burden and hope among patients with inflammatory bowel disease. Nurs. Open.

[31] Pakdin M., Zarei L., Bagheri Lankarani K., Ghahramani S. (2023). The cost of illness analysis of inflammatory bowel disease. BMC Gastroenterol..

[32] Magro F., Portela F., Lago P., Deus J., Cotter J., Cremers I., Vieira A., Peixe P., Caldeira P., Lopes H. (2009). Inflammatory bowel disease: A patient’s and caregiver’s perspective. Dig. Dis. Sci..

[33] Napolitano D., Vellone E., Iovino P., Scaldaferri F., Cocchieri A. (2024). Self-care in patients affected by inflammatory bowel disease and caregiver contribution to self-care (IBD-SELF): A protocol for a longitudinal observational study. BMJ Open Gastroenterol..

[34] Napolitano D., Biagioli V., Bartoli D., Cilluffo S., Martella P., Monaci A., Vellone E., Cocchieri A. (2025). Validity and Reliability of the Self-Care of Chronic Illness Inventory in Patients Living with Inflammatory Bowel Disease. J. Clin. Nurs..

[35] De Maria M., Iovino P., Lorini S., Ausili D., Matarese M., Vellone E. (2021). Development and Psychometric Testing of the Caregiver Self-Efficacy in Contributing to Patient Self-Care Scale. Value Health.

[36] Yu D.S.-F., De Maria M., Barbaranelli C., Vellone E., Matarese M., Ausili D., Rejane R.-S.E., Osokpo O.H., Riegel B. (2021). Cross-cultural applicability of the Self-Care Self-Efficacy Scale in a multi-national study. J. Adv. Nurs..

[37] Coates M.D., Binion D.G. (2021). Silent Inflammatory Bowel Disease. Crohns Colitis 360.

[38] Gonczi L., Bessissow T., Lakatos P.L. (2019). Disease monitoring strategies in inflammatory bowel diseases: What do we mean by “tight control”?. World J. Gastroenterol..

[39] Dolinger M., Torres J., Vermeire S. (2024). Crohn’s disease. Lancet.

[40] Musters S.C.W., Kreca S.M., van Dieren S., van der Wal-Huisman H., Romijn J.A., Chaboyer W., Nieveen van Dijkum E.J.M., Eskes A.M., ARTIS Consortium (2024). Family caregiver outcomes after participating in a hospital-based family involvement program after major gastrointestinal surgery: A subgroup analysis of a patient preferred cohort study. Int. J. Surg..

[41] DuBois K.E., Blake C.E., Rudisill C., Harrison S.E., Hébert J.R. (2024). Use of Treatment and Self-Management Methods: Perspectives and Decisions of Patients with Ulcerative Colitis. Am. J. Lifestyle Med..

[42] Buck H.G., Harkness K., Wion R., Carroll S.L., Cosman T., Kaasalainen S., Kryworuchko J., McGillion M., O’Keefe-McCarthy S., Sherifali D. (2015). Caregivers’ contributions to heart failure self-care: A systematic review. Eur. J. Cardiovasc. Nurs..

[43] Matarese M., Pendoni R., Piredda M., De Marinis M.G. (2021). Caregivers’ experiences of contributing to patients’ self-care in Chronic Obstructive Pulmonary Disease: A thematic synthesis of qualitative studies. J. Adv. Nurs..

[44] Choi J.Y., Lee S.H., Yu S. (2024). Exploring Factors Influencing Caregiver Burden: A Systematic Review of Family Caregivers of Older Adults with Chronic Illness in Local Communities. Healthcare.

[45] Suresh M., Young J., Fan V., Simons C., Battaglia C., Simpson T.L., Fortney J.C., Locke E.R., Trivedi R. (2021). Caregiver Experiences and Roles in Care Seeking During COPD Exacerbations: A Qualitative Study. Ann. Behav. Med..

[46] Pacheco Barzallo D., Schnyder A., Zanini C., Gemperli A. (2024). Gender Differences in Family Caregiving. Do female caregivers do more or undertake different tasks?. BMC Health Serv. Res..

[47] Fiorino G., Caprioli F.A., Onali S., Macaluso F.S., Bezzio C., Armelao F., Armuzzi A., Baldoni M., Bodini G., Castiglione F. (2025). Adaptation of the European Crohn’s Colitis Organisation quality of care standards to Italy: The Italian Group for the study of inflammatory bowel disease consensus. Dig. Liver Dis..

[48] Napolitano D., Vincenzo F.D., Orgiana N., Schiavoni E., Germini F., Pugliese D., Scaldaferri F., IBD UNIT-CEMAD (2024). The inflammatory bowel disease care manager: Italian state of the art. Ann. Gastroenterol..

