# Supplementary material for: Caregiver Contribution to Self-Care in Adults with Inflammatory Bowel Disease: A Cross-Sectional Multicenter Study
Source: Nurs Rep. 2026 Mar 27;16(4):110. doi: 10.3390/nursrep16040110 (PMC13118427; doi:10.3390/nursrep16040110)
Supplement: Supplementary file 1 [file nursrep-16-00110-s001.zip › nursrep-4184104-supplementary.pdf]

STROBE Statement—checklist of items that should be included in reports of observational studies

	Item No.	Recommendation	Page No.	Relevant text from manuscript
**Title and abstract**	1	(*a*) Indicate the study's design with a commonly used term in the title or the abstract	Title; Abstract	Caregiver Contribution to Self-Care in Adults with Inflammatory Bowel Disease: A Cross-Sectional Multicenter Study
		(*b*) Provide in the abstract an informative and balanced summary of what was done and what was found	Abstract	Inflammatory bowel disease (IBD) requires sustained self-care, yet patients' ability to manage daily treatment and symptoms is often shaped by the support provided by informal caregivers.
**Introduction**				
Background/rationale	2	Explain the scientific background and rationale for the investigation being reported	Introduction	Inflammatory Bowel Disease (IBD) is a chronic, relapsing, remitting disorder comprising Crohn's Disease (CD) and Ulcerative Colitis (UC) that imposes substantial burdens on patients and healthcare systems worldwide [1].
Objectives	3	State specific objectives, including any prespecified hypotheses	End of Introduction	Although specific research on caregivers' roles in IBD remains limited, early evidence suggests that caregiver support is crucial for improving treatment adherence, dietary management, and early symptom recognition.
**Methods**				

Study design	4	Present key elements of study design early in the paper	Methods 2.1 Design	A multicenter, cross-sectional study was conducted between April and June 2024.
Setting	5	Describe the setting, locations, and relevant dates, including periods of recruitment, exposure, follow-up, and data collection	Methods 2.2 Setting and Sampling; 2.1 Design	A convenience sample was collected from consecutive caregivers of outpatient patients diagnosed with IBD (CD and UC).
Participants	6	(*a*) *Cohort study*—Give the eligibility criteria, and the sources and methods of selection of participants. Describe methods of follow-up<br>*Case-control study*—Give the eligibility criteria, and the sources and methods of case ascertainment and control selection. Give the rationale for the choice of cases and controls<br>*Cross-sectional study*—Give the eligibility criteria, and the sources and methods of selection of participants	Methods 2.2 Inclusion/Exclusion Criteria	Eligible participants were primary caregivers of adult patients with a confirmed diagnosis of CD or UC. Caregivers were required to be 18 years or older and to have provided care for at least six months.
		(*b*) *Cohort study*—For matched studies, give matching criteria and number of exposed and unexposed<br>*Case-control study*—For matched studies, give matching criteria and the number of controls per case	N/A	Not applicable (no matching performed in this cross-sectional study).
Variables	7	Clearly define all outcomes, exposures, predictors, potential confounders, and effect modifiers. Give diagnostic criteria, if applicable	Methods 2.3 Instruments	Caregivers completed a structured questionnaire collecting data on age, gender, education, marital status, employment status, relationship to the patient, living arrangements, caregiving experience, and use of support services and patient fatigue attention, defined as the caregiv…
Data sources/ measurement	8*	For each variable of interest, give sources of data and details of methods of assessment (measurement). Describe comparability of assessment methods if there is more than one group	Methods 2.4–2.5 (CC-SC-CII; CSE-CSC)	Caregiver contribution to self-care was assessed using the Caregiver Contribution to Self-Care of Chronic Illness Inventory (CC-SC-CII) [24,43,44], a 19-item psychometrically sound instrument that evaluates the extent to which

				caregivers support patients across the three componen…
Bias	9	Describe any efforts to address potential sources of bias	Methods 2.2 Setting and Sampling	A convenience sample was collected from consecutive caregivers of outpatient patients diagnosed with IBD (CD and UC).
Study size	10	Explain how the study size was arrived at	Not reported	Study size justification or sample size calculation was not reported in the current manuscript.

Continued on next page

Quantitative variables	11	Explain how quantitative variables were handled in the analyses. If applicable, describe which groupings were chosen and why	Methods 2.6 Statistical analysis	All statistical analyses were conducted using R software, version 4.3.3, along with relevant R packages for data management, visualization, and statistical modelling. Descriptive analyses were conducted to summarize the characteristics of the sample.
Statistical methods	12	(*a*) Describe all statistical methods, including those used to control for confounding	Methods 2.6 Statistical analysis	All statistical analyses were conducted using R software, version 4.3.3, along with relevant R packages for data management, visualization, and statistical modelling. Descriptive analyses were conducted to summarize the characteristics of the sample.
		(*b*) Describe any methods used to examine subgroups and interactions	Methods 2.6 Statistical analysis	Linear regression models were used to examine associations between caregiver- and patient-related predictors and the three domains of the CC-SC-CII self-care instrument: Maintenance, Monitoring, and Management.
		(*c*) Explain how missing data were addressed	Not reported	Handling of missing data was not described in the current manuscript.
		(*d*) *Cohort study*—If applicable, explain how loss to follow-up was addressed<br>*Case-control study*—If applicable, explain how matching of cases and controls was addressed<br>*Cross-sectional study*—If applicable, describe analytical methods taking account of sampling strategy	N/A	Not applicable (no complex sampling strategy was used).
		(*e*) Describe any sensitivity analyses	Not reported	Sensitivity analyses were not reported in the current manuscript.
Participants	13*	(a) Report numbers of individuals at each stage of study—eg numbers potentially eligible, examined for eligibility, confirmed eligible, included in the study, completing follow-up, and analysed	Results (opening paragraph)	A total of 275 caregivers were enrolled in the study. The study is evenly split between caregivers of patients with CD

				(47.6%) and patients with UC (52.4%), with a median age of 43.5 years.
		(b) Give reasons for non-participation at each stage	Not reported	Reasons for non-participation were not reported in the current manuscript.
		(c) Consider use of a flow diagram	Not reported	A participant flow diagram was not included in the current manuscript.
Descriptive data	14*	(a) Give characteristics of study participants (eg demographic, clinical, social) and information on exposures and potential confounders	Results; Table 1	A total of 275 caregivers were enrolled in the study. The study is evenly split between caregivers of patients with CD (47.6%) and patients with UC (52.4%), with a median age of 43.5 years.
		(b) Indicate number of participants with missing data for each variable of interest	Not reported	Numbers of participants with missing data for each variable were not reported in the current manuscript.
		(c) *Cohort study*—Summarise follow-up time (eg, average and total amount)	N/A	Not applicable (cross-sectional study; no follow-up time).
Outcome data	15*	*Cohort study*—Report numbers of outcome events or summary measures over time	Results; Table 2	The total CC-SC-CII Maintenance score was 68.0 [44.5–79.0] for the caregivers' group of CD patients and 52.0 [32.0–71.0] for the caregivers' group of UC patients. The total CC-SC-CII Monitoring score was 80.0 [55.0–97.5] for the group CD and 75.0 [45.0–95.0] for the group.
		Case-control study—Report numbers in each exposure category, or summary measures of exposure	Results; Table 2	The total CC-SC-CII Maintenance score was 68.0 [44.5–79.0] for the caregivers' group of CD patients and 52.0 [32.0–71.0] for the caregivers' group of UC patients. The total CC-SC-CII Monitoring score was 80.0 [55.0–97.5] for

				the group CD and 75.0 [45.0–95.0] for the group.
		Cross-sectional study—Report numbers of outcome events or summary measures	Results; Table 2	The total CC-SC-CII Maintenance score was 68.0 [44.5–79.0] for the caregivers' group of CD patients and 52.0 [32.0–71.0] for the caregivers' group of UC patients. The total CC-SC-CII Monitoring score was 80.0 [55.0–97.5] for the group CD and 75.0 [45.0–95.0] for the group.
Main results	16	(*a*) Give unadjusted estimates and, if applicable, confounder-adjusted estimates and their precision (eg, 95% confidence interval). Make clear which confounders were adjusted for and why they were included	Results; Table 4	Note. Values are reported as B (SE, p-value); CC-SC-CII, Caregiver Contribution to Self-Care of Chronic Illness Inventory; CD= Crohn's Disease, UC=Ulcerative Colitis.
		(*b*) Report category boundaries when continuous variables were categorized	Methods 2.6 Statistical analysis	Predictors were included either as continuous or categorical variables, depending on their nature. Some categorical predictors with more than two levels were dichotomized to improve model interpretability and reduce overfitting, particularly when certain levels had sparse data.
		(*c*) If relevant, consider translating estimates of relative risk into absolute risk for a meaningful time period	N/A	Not applicable (continuous outcomes; no relative risks reported).

Continued on next page

Other analyses	17	Report other analyses done—eg analyses of subgroups and interactions, and sensitivity analyses	Methods 2.6; Results (bivariate and regression analyses)	Preliminary bivariate associations were explored to further characterize the relationships among study variables. Point-biserial correlations were computed to examine the associations between continuous outcomes and dichotomous variables.
**Discussion**				
Key results	18	Summarise key results with reference to study objectives	Discussion (opening paragraph)	This multicentre cross-sectional study provides new descriptive insights into the contribution of informal caregivers to self-care in IBD patients, highlighting significant differences between CD and UC and patterns of association with selected sociodemographic and caregiving-rel…
Limitations	19	Discuss limitations of the study, taking into account sources of potential bias or imprecision. Discuss both direction and magnitude of any potential bias	Discussion 4.1 Strengths and Limitations	This study's strengths include its multicentre design, relatively large and balanced sample, and the use of validated tools (CC-SC-CII and CSE-CSC).
Interpretation	20	Give a cautious overall interpretation of results considering objectives, limitations, multiplicity of analyses, results from similar studies, and other relevant evidence	Discussion 4; Conclusions	Regression models further illustrated these patterns by identifying pathology-specific associations.
Generalisability	21	Discuss the generalisability (external validity) of the study results	Discussion 4.1 (partial); Not explicitly addressed	Generalisability (external validity) was not explicitly discussed; the sample comprised caregivers of adult outpatients recruited across multiple Italian centres.
**Other information**				
Funding	22	Give the source of funding and the role of the funders for the present study and, if applicable, for the original study on which the present article is based	Funding	Funding: This research received no external funding

*Give information separately for cases and controls in case-control studies and, if applicable, for exposed and unexposed groups in cohort and cross-sectional studies.

Note: An Explanation and Elaboration article discusses each checklist item and gives methodological background and published examples of transparent reporting. The STROBE checklist is best used in conjunction with this article (freely available on the Web sites of PLoS Medicine at http://www.plosmedicine.org/, Annals of Internal Medicine at http://www.annals.org/, and Epidemiology at http://www.epidem.com/). Information on the STROBE Initiative is available at www.strobe-statement.org.
